# Blood-Derived α-Synuclein Aggregated in the Substantia Nigra of Parabiotic Mice

**DOI:** 10.3390/biom11091287

**Published:** 2021-08-29

**Authors:** Xizhen Ma, Leilei Chen, Ning Song, Le Qu, Jun Wang, Junxia Xie

**Affiliations:** Shandong Provincial Key Laboratory of Pathogenesis and Prevention of Neurological Disorders, Institute of Brain Science and Disease, Qingdao University, Qingdao 266071, China; 17806287523@163.com (X.M.); leileichen2019@qdu.edu.cn (L.C.); ningsong@qdu.edu.cn (N.S.); ql871118@163.com (L.Q.)

**Keywords:** Parkinson’s disease, alpha-synuclein, transmission, A53T, parabiosis

## Abstract

As a pathological biomarker of Parkinson’s disease, α-synuclein is thought to be a prion-like protein, but evidence for the transmission of α-synuclein from blood to the brain is unclear. The goals of this study were to determine whether blood-derived α-synuclein could enter the brains of mice and whether α-synuclein in the brain could be cleared by parabiosis. Heterochronic parabiosis was performed on *SNCA*^A53T^ transgenic mice (A53T mice) and wildtype mice. The levels of human α-synuclein in the blood and substantia nigra of wildtype mice were significantly increased after 4-month parabiosis with A53T mice. Moreover, the expression of α-synuclein filament, but not of total α-synuclein, was significantly increased in the substantia nigra of wildtype mice that were paired with A53T mice. However, the levels of human α-synuclein displayed no significant change in the serum, blood, or substantia nigra of A53T mice. These results provide direct evidence that pathological α-synuclein can be transmitted from blood to the brain in the heterochronic parabiosis system; however, it appears to be difficult to clear it from the brain in a short period of time.

## 1. Introduction

As the most important biomarker of Parkinson’s disease and a major component of Lewy bodies, α-synuclein is thought to be a prion-like protein [1]. It has been reported that abnormal tangling and increased aggregation of α-synuclein are strongly associated with dopaminergic neuron damage in the substantia nigra (SN) of patients with Parkinson’s disease [2,3,4], and therefore, the spread and elimination of α-synuclein have become hot topics in neuroscience [5,6]. Independent epidemiological datasets have revealed that appendix removal is associated with a lower risk of Parkinson’s disease, and the appendix contains pathology-associated α-synuclein [7]. Full truncal vagotomy also decreases the risk [8]. If labelled pathological α-synuclein is injected into the intestinal nerve, the fluorescent labels can be observed in the brain [9]. Although the hypothesis of pathology seeds has been well discussed in the past two decades [10,11], most studies on it have been confined to α-synuclein transmission in the central nervous system. Moreover, direct evidence regarding whether blood-derived α-synuclein could enter the brain is limited. It has been reported that erythrocyte-derived α-synuclein-rich extracellular vesicles can cross the blood–brain barrier (BBB) via adsorptive-mediated transcytosis and are then ingested by microglia cells [12]. However, that study did not determine whether pathological α-synuclein can continuously exist in the central nervous system, as it is limited by low levels of α-synuclein.

Recently, a controversial conclusion was reported regarding parabiosis between C57BL/6J mice injected with Lewy body fractions in the SN and normal mice with patient-derived α-synuclein—that is, the latter not being “transmitted” through the bloodstream within 4-month parabiosis [13].

It has been reported that the overexpression of mutated A53T human α-synuclein (Hu-α-syn) via a viral vector in the SN of mice, rats, and marmosets can cause dopaminergic neuron lesions [14,15,16]. As there are seven amino acids that differ between mouse α-synuclein and Hu-α-syn [17], specific antibodies were constructed and ELISA was employed to identify the level of Hu-α-syn.

In this study, heterochronic parabiosis surgery was performed on *SNCA*^A53T^ transgenic mice (A53T mice), which overexpressed mutant Hu-α-syn, and wildtype (WT) mice in the same litter. ELISA with a specific antibody was employed to identify whether the Hu-α-syn produced in A53T mice could enter the bloodstream of WT mice and then accumulate in the substantia nigra of WT mice.

## 2. Materials and Methods

### 2.1. Animals

All procedures were carried out in accordance with the National Institutes of Health Guide for the Care and Use of Laboratory Animals and were approved by the Ethical Committee of the Medical College of Qingdao University (Ethical approval number: 2020031601, date: 16 March 2020). *SNCA*^A53T^ transgenic mice (A53T mice) that overexpress human mutant α-synuclein were purchased from the Jackson Laboratory (Bar Harbor, ME, USA). The mutant gene expression was quantified by real-time PCR as previously described [18]. A53T male mice with ΔCt values of 4.5–5.5 and WT mice were bred by in vitro fertilization for parabiotic surgery in Shanghai Model Organisms Center (Shanghai, China). In total, 57 mice (8 weeks old, 20–25 g), including 28 A53T mice and 29 WT mice, were used in this study. The animals were maintained at 23 ± 2 °C under a 12-h light/dark cycle. Air humidity was approximately 50%. Mice were housed in standard laboratory cages. Food and water were freely available. Sodium pentobarbital was used to anesthetize mice.

### 2.2. Immunofluorescence (IF)

Mice were transcardially perfused with normal saline after being deeply anesthetized with sodium pentobarbital and then perfused with 4% paraformaldehyde (PFA). Brains were postfixed in 4% PFA overnight and then dehydrated with 20% and 30% sucrose successively (dissolved in 0.1 M PBS). The 12 adjacent frozen brain slices (20 μm thick; section was from bregma–2.92 mm to bregma–3.16 mm) of the same coronal section were selected in strict accordance with the mouse brain atlas. IF staining was used to detect the expression of α-synuclein, TH, and α-synuclein filament (fila-α-syn) in the SN of 6-month-old mice.

We used 5% donkey serum (Jackson, 017-000-121) in PBST (0.3% Trition-100 in PBS) to block non-specific binding sites (room temperature, 30 min). Primary antibodies against TH (chicken, abcam, ab76442, 1:3000 dilution), α-synuclein (rabbit, abcam, ab155038, 1:200 dilution), and fila-α-syn (rabbit, abcam, ab209538, 1:5000 dilution) were used at 4 °C overnight. Appropriate secondary antibodies were then incubated at room temperature (1:500 dilution) for 2 h. A digital pathological section scanning system (OLYMPUS, Tokyo, Japan, VS120) was used to take pictures.

Three 20 μm thick brain slices (at 60 μm intervals) were chosen from the section of bregma–2.92 mm to–3.16 mm. TH-positive cells were stereologically counted in the SN, and the mean of the unilateral cell count was used to represent the number of dopaminergic neurons. Randomized counting frames were used for α-synuclein-positive cells in the SN; the average of the cell counts in each frame was used to represent the expression of α-synuclein. The integrated optical density of fila-α-syn-positive signaling in the SN was used as the measure of fila-α-syn content.

### 2.3. Parabiosis Surgery

**Figure 1:**
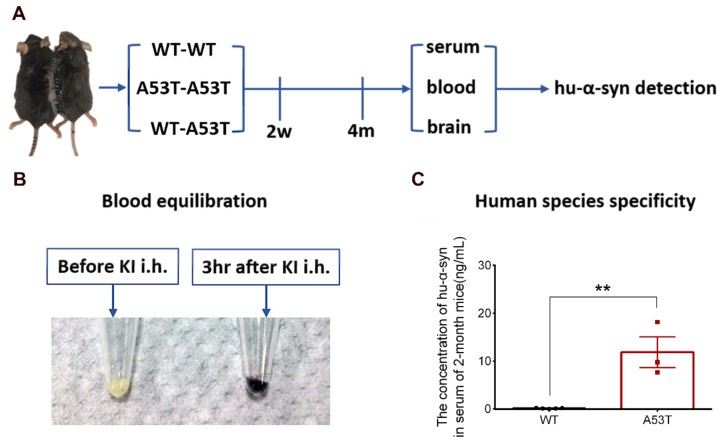
Experimental design and analysis of feasibility. (**A**) Homologous WT-A53T parabiosis group and WT-WT or A53T-A53T sham-operated group mice were surgically joined together. (**B**) Two weeks after the operation, blood equilibration was observed with KI injection. (**C**) No Hu-α-syn was detected in serum of WT mice, while serum of A53T mice contains a large amount of Hu-α-syn. (*n* = 5, 3, ** *p* < 0.01, the specific levels of each sample in different groups were shown as “•” and “▪”).

The parabiosis surgery protocol followed literature reports [19]. The identification of genotypes was achieved in real-time PCR. Eight-week-old heterozygous A53T mice and homologous WT mice received this surgery. The parabiosis surgery was performed in a clean animal surgery room, and autoclaved surgical instruments were prepared before surgery. Mice were anesthetized with sodium pentobarbital. Skin preparation was necessary for the sutured side. A longitudinal skin incision on a shaved side of each animal was made from 0.5 cm above the elbow to 0.5 cm below the knee joint using sharp scissors after sufficient pre-operative skin preparation. The fascia was separated to create 0.5 cm of free skin; olecranons, knee joints and skin were sewn together in turn. In the parabiosis group, one homologous WT mouse was paired with each A53T mouse (WT–A53T, 9 pairs). At the same time, in the sham-operated group, homologous WT–WT (4 pairs) and A53T–A53T (5 pairs) combinations were used (Figure 1A). Each mouse was intraperitoneally injected with penicillin (50,000 units per day) to prevent infection for three days after surgery. Oral ibuprofen was given in their water (0.02 mg/mL) within 2 weeks after surgery if necessary. Each pair of mice was kept in a single cage after surgery.

### 2.4. Equilibration of the Blood of Parabiotic Partners

With the purpose of determining whether mouse blood equilibration was successfully fulfilled 2 weeks after surgery, hypodermic injection potassium iodide (KI) at the back of the left parabiotic mice at a dose of 0.15 mL (250 mg/mL). Urine from the right one was collected both before and 3 h after the injection. A total of 30% H_2_O_2_ was added to the urine to change the I^−^ to I_2_. Then, the oxidized urine was added to starch. Iodide ions in the blood enter the urine and are oxidized to I_2_ by H_2_O_2_. I_2_ can make starch turn blue. (Figure 1B).

### 2.5. ELISA Test of Hu-α-syn

The levels of Hu-α-syn in the serum, blood, and brain tissue were detected before surgery and 4 months after surgery. Mouse caudal vein blood samples were collected from deeply anesthetized mice and were then divided into two tubes. One of the tubes was quickly dropped into liquid nitrogen for quick-freezing; the other was left at room temperature for 30 min followed by centrifugation at 3000 rpm after clotting. The supernatant (serum) was sucked into new tubes. Mouse brains were quickly removed from skulls after being transcardially perfused with normal saline and sliced into 1-mm-thick (bregma −2.80–−3.80 mm) slices on an ice-cold Rodent Brain Matrix (RBM-2000C, ASI Instruments, Amsterdam, The Netherlands). The SN was separated from the slices according to the mouse brain atlas. The SN samples were lysed with RIPA lysis buffer (25 uL/mg) (CW2333, CWBIO, Beijing, China). The harvested lysate was centrifuged at 12,000× *g* for 30 min at 4 °C, and the supernatant was used for analysis. The Hu-α-syn ELISA Kit was provided by BioLegend (844101, San Diego, CA, USA). Hu-α-syn could be specifically identified by this ELISA kit (Figure 1C).

### 2.6. Western Blotting

The bicin-choninic Acid (BCA) Protein Assay Kit (CW2011S, CWBIO, Beijing, China) was used to calculate protein concentration. The loading buffer was added to the protein supernatant, which was collected as described in the ELISA test protocol, heated for 5 min (95 °C), loaded on 10% acrylamide–SDS gel (P0056B, Shanghai, China), and transferred to a polyvinylidene fluoride (PVDF) membrane (Millipore, Billerica, MA, USA). The molecular weight of the protein was marked by a protein marker (26616, Thermo, Waltham, MA, USA). The membrane was blocked with 5% skim milk in TBST for 2 h at room temperature. Primary antibodies anti-α-synuclein (Abcam, ab155038, Cambridge, UK), anti-olig2 (CST, 17198, Boston, MA, USA), anti-Iba1 (CST, ab109186, Boston, MA, USA), and anti-β-actin (Bioss, bs-0061R, Beijing, China) were incubated overnight at 4 °C and washed. Species-appropriate, HRP-conjugated secondary antibodies were incubated for 1 h at room temperature and washed three times. Enhanced chemiluminescence reagents reacted with HRP, and the signal intensity value was read by the ultra-sensitive chemiluminescence system (Fusion FX.EDGE, VILBER Smart Imaging, Paris, France). The integrated density ratio of α-synuclein: β-actin represents the expression level of α-synuclein, as do the olig2 and Iba1.

### 2.7. Dot Blot

Protein samples were collected as described above. The total protein concentration in these supernatant samples were detected using a BCA assay kit as described above. The protein supernatant was diluted with PBS to 2 μg/μL. Then, 10 μg supernatant was dropped onto the nitrocellulose (NC) filter membrane without loading buffer or heating. The membrane was blocked with 5% skim milk in TBST for 1 h at room temperature. Primary antibody anti-α-synuclein filament (abcam, ab209538, Cambridge, UK) was incubated for 1 h at room temperature and washed three times with TBST. Goat anti-rabbit HRP-conjugated secondary antibody (absin, abs20002, Shanghai, China) was incubated for 1 h at room temperature and washed three times. The imaging system was the same as that used for Western blotting. The integrated density values of fila-α-syn represent the qualitative changes in fila-α-syn content.

### 2.8. Statistical Analysis

The integrated optical densities of immunofluorescence images, the gray-scale values of Western blotting, and dot blots were analyzed using ImageJ (1.6.0, NIH, Bethesda, MD, USA). The results were analyzed using SPSS 23.0 statistical software (IBM, CHI, Amongk, NY, USA) and are expressed as means ± standard errors of the mean ($\bar{x}$ ± S.E.M.). A comparison of the two groups was performed using an independent sample *t*-test; a comparison of multiple groups was made using one-way ANOVA; and the Student–Newman–Keuls test was used to perform a comparison of the means of the two groups.

## 3. Results

### 3.1. Experimental Design and Feasibility Evaluation

**Figure 2:**
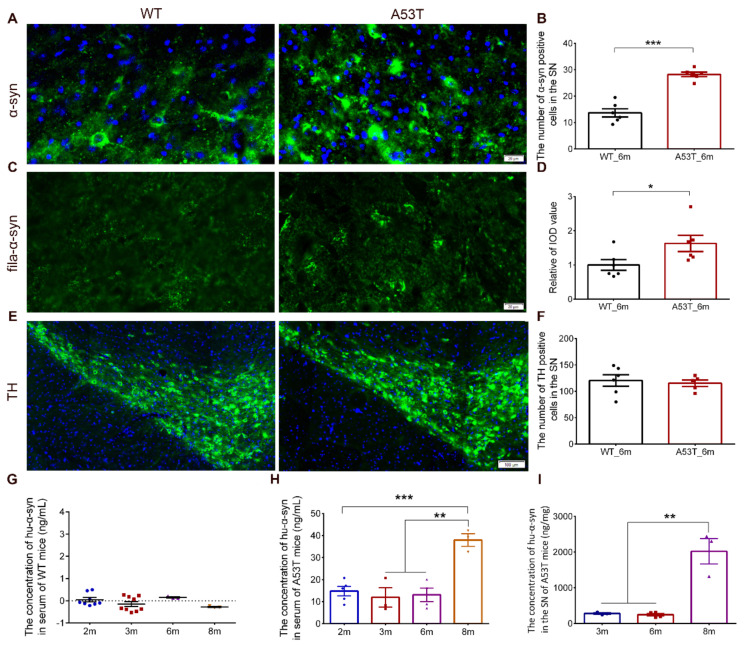
The pathologic changes in α-synuclein of A53T mice. (**A**,**B**) The number of α-synuclein positive cells (green) in the SN of A53T mice was up-regulated compared with the WT mice. (*n* = 6, *** *p* < 0.001, nucleus were labeled with blue DAPI, bar = 20 μm). (**C**,**D**) The integrated optical density (IOD) of α-synuclein filament (fila-α-syn, green) in the SN of A53T mice was up-regulated significantly compared with the WT mice. (*n* = 6, * *p* < 0.05, bar = 20 μm). (**E**,**F**) No changes was found in the number of TH positive cells (green) in the SN. (*n* = 6, 5, nucleus were labeled with blue DAPI, bar = 100 μm). (**G**) No Hu-α-syn was detected in serum of WT mice at all time points. (**H**,**I**) The concentration of Hu-α-syn in the serum and SN of A53T mice was stable before 6 months and increased sharply in 8 months A53T mice. (*n* ≥ 3, ** *p* < 0.01, *** *p* < 0.001). (The specific levels of each sample in different groups were shown as “•”, “▪”, “▲”, and ”▼”).

Immunofluorescence images revealed that both the number of α-synuclein positive cells and the integrated optical density of fila-α-syn were significantly increased in the SN of 6-month-old A53T mice compared to the SN of WT mice (Figure 2A–D). No significant difference in TH-positive cell count was found between the A53T mice and WT mice within the framework we examined (Figure 2E,F). As existing research reported, no dopaminergic neuron loss in substantia nigra of A53T mice was found until 10 months old [20], which is consistent with our result. In addition, Hu-α-syn was not found in the serum of WT mice of any age group (Figure 2G), indicating that Hu-α-syn was specifically detected with the ELISA kit with a specific antibody. The levels of Hu-α-syn in the serum and SN were stable until the A53T mice were 6 months old; said levels sharply increased in the group of 8-month-old A53T mice (Figure 2H,I). In order to avoid the interference caused by Hu-α-syn generation after the age of 6 months, parabiosis surgery was performed on the 2-month-old mice, and the time period of parabiosis was set to 4 months.

In the parabiosis group, homologous WT mice were paired with 2-month-old A53T mice (WT–A53T), whereas in the sham-operated group, homologous WT–WT or A53T–A53T were used (Figure 1A). After two weeks, the blood equilibration of parabiotic mice was evaluated. The KI was injected in the left mouse (i.h.), and urine was collected from the right mouse and oxidized with H_2_O_2_. In the group subjected to KI injection, the oxidized urine blued the starch (Figure 1B). In addition, the concentration of Hu-α-syn in the serum of 2-month-old A53T mice was significantly higher than that in the WT mice, and no Hu-α-syn was found in the serum of WT mice (Figure 1C).

### 3.2. Alpha-Synuclein Pathology Was Significantly Increased in the SN of Parabiotic WT Mice

**Figure 3:**
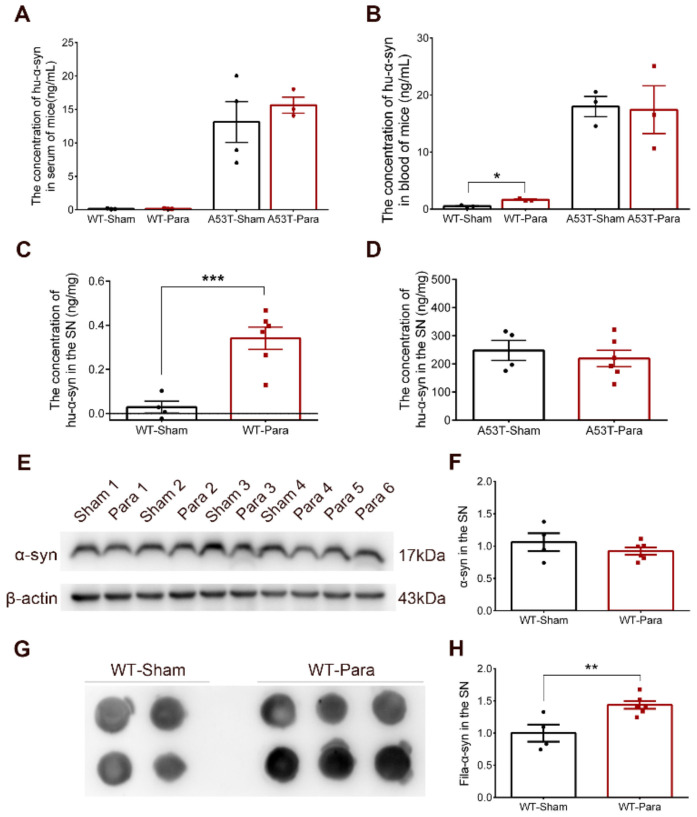
The pathological changes of α-synuclein in the SN of WT mice after parabiosis. (**A**) No Hu-α-syn was detected in the serum of parabiotic WT mice and no change was found in the concentration of Hu-α-syn in the serum of A53T mice (*n* = 3). (**B**) The concentration of Hu-α-syn in the blood was up-regulated in parabiotic WT mice after 4-month parabiosis, and no change was found in the concentration of Hu-α-syn in the blood of parabiotic A53T mice (*n* = 3, * *p* < 0.05). (**C**) The concentration of Hu-α-syn in the SN of parabiotic WT mice was up-regulated after 4-month parabiosis (*n* = 4, 6, *** *p* < 0.001). (**D**) No change was found in the expression of Hu-α-syn in the SN of parabiotic A53T mice (*n* = 4, 6). (**E**,**F**) No change was found in the expression of total α-synuclein (α-syn) in the SN of parabiotic WT mice (*n* = 4, 6). (**G**,**H**) The fila-α-syn in the SN of parabiotic WT mice was significantly increased after 4-month parabiosis with A53T mice (*n* = 4, 6, ** *p* < 0.01). (The specific levels of each sample in different groups were shown as “•” and “▪”).

The levels of Hu-α-syn in the serum and blood were detected after 4-month parabiosis. No significant changes in Hu-α-syn were found in the sera of parabiotic WT and A53T mice, or in the blood of parabiotic A53T mice (Figure 3A,B). Interestingly, the levels of Hu-α-syn in the blood and SN were significantly increased in the WT mice, which were paired with A53T mice for 4 months (Figure 3B,C). No changes in Hu-α-syn levels were found in the SN of parabiotic A53T mice when compared with the sham group (Figure 3D). Heterochronic parabiosis abortively decreased the content of Hu-α-syn in the brains of A53T mice. The upregulation of Hu-α-syn in the blood of parabiotic WT mice explained the origin of Hu-α-syn in the brains of WT mice, although the Hu-α-syn from A53T mice was eliminated in the sera of WT mice. Although no difference was found in the expression level of total α-synuclein in the SN (Figure 3E,F), the fila-α-syn in the SN of WT mice was significantly increased after 4-month parabiosis with A53T mice (Figure 3G,H). Furthermore, no significant changes in Iba1 and olig2 were found in the SN of parabiotic WT mice (Figure S1A–C).

## 4. Discussion

It has been reported that the aggregation propensities of mutant α-synuclein and the neurotoxicity of oligomers are related to the pathogenesis of PD [2,21]. Misfolded α-synuclein can be transmitted in exosomes; however, discrepancies in pathological results among different studies, due to the concentrations of exosomes injected, caused concern [22,23]. Monomeric α-synuclein disrupted the BBB by interacting with pericytes in patients with PD [24]. Oligomers, ribbons, and fibrils α-synuclein have also been demonstrated to cross the BBB and enter the brain after intravenous administration in mice [25]. A study based on radioactively labeled α-synuclein provided direct evidence that α-synuclein can cross the BBB in both the brain-to-blood and the blood-to-brain directions [26]. However, it is still controversial whether pathologic α-synuclein can cross the BBB from blood to the brain. A study on parabiosis has identified human Aβ in the brains of WT mice, which provided direct evidence that Aβ seeds circulating in blood can enter the central nervous system after 12-month parabiosis [27]. Therefore, we established a new parabiotic model of A53T and WT mice in this study. Iodide circulated from the left mouse to the right mouse and was excreted via urine after 3 h injection, indicating that blood equilibration was successfully established after 2 weeks of parabiosis.

After 4 months of parabiosis, Hu-α-syn appeared in the blood and SN, but not in sera of the WT mice that were paired with A53T mice, indicating that exogenous Hu-α-syn from A53T mice may be cleared in the sera of WT mice, but preserved in membrane structure [12], where they may increase the BBB permeability (similar observations have been made in PD patients), and finally be transported across the BBB, rather than via afferent nerve fibers in peripheral organs. We also observed that a recently published study came to a different conclusion in regard to parabiotic mice injected with Lewy body fractions in the SN paired with normal mice [13]. Their Lewy body stereotactic injection was carried out on the same day as the parabiosis surgery, and as such, the pathological changes produced by the Lewy body injection could not affect parabiotic mice during the early stage. Moreover, this study tested plasma, but did not rule out the role of pathological protein components that were protected by membrane structures, such as blood cell membranes. These may be the reasons for the different conclusions. In addition, in our research, no changes in Hu-α-syn were observed in the SN of A53T mice after 4 months of parabiosis, and it appeared to be difficult to clear pathologic α-synuclein in the brain, which may be due to the impairment of the autophagy-lysosome pathway [28,29,30,31].

Our study still has some limitations. Firstly, the antibody used to detect fila-α-syn was not human-specific, whether the increased fila-α-syn detected by dot blot was endogenous aggregation or exogenous Hu-α-syn remains further discussion. In addition, the levels of Hu-α-syn in the SN of WT-para mice were less than 0.5 ng/mg, immunostaining or capillary depletion is helpful to demonstrate that Hu-α-syn entered brain parenchyma [12]. Based on the low concentration of Hu-α-syn in the blood of WT-para mice (1.65 ng/mL) and sufficient cardiac perfusion, we tend to think that Hu-α-syn entered brain parenchyma.

Although our research was preliminary, it was suggestive and informative, strongly indicating that α-synuclein could be transmitted from blood to the brain, and the transmission was not in the form of free proteins in serum. Further studies on tracers and mechanisms, and studies on precisely how Hu-α-syn crosses the BBB from peripheral blood circulation into the brain, are urgently required. Taken together, our results provide direct evidence that pathological α-synuclein could cross the BBB and be transmitted from blood to the brain. Importantly, this study was the first report regarding the parabiotic mouse model of A53T and WT mice, which may serve as a good in vivo model for the investigation of prion-like transmission of α-synuclein from blood to the brain, especially for the screening of potential drugs or molecular targets to prevent PD development.

## Data Availability

The datasets are available from the corresponding author on reasonable request.

## Supplementary Materials

The following are available online at https://www.mdpi.com/article/10.3390/biom11091287/s1, Figure S1: The levels of Iba1 and olig2 in the SN of WT mice after parabiosis.
