# Supplementary material for: Blood-Derived α-Synuclein Aggregated in the Substantia Nigra of Parabiotic Mice
Source: Biomolecules. 2021 Aug 29;11(9):1287. doi: 10.3390/biom11091287 (PMC8471402; doi:10.3390/biom11091287)
Supplement: Supplementary file 1 [file biomolecules-11-01287-s001.zip › biomolecules-1311030-supplementary.pdf]

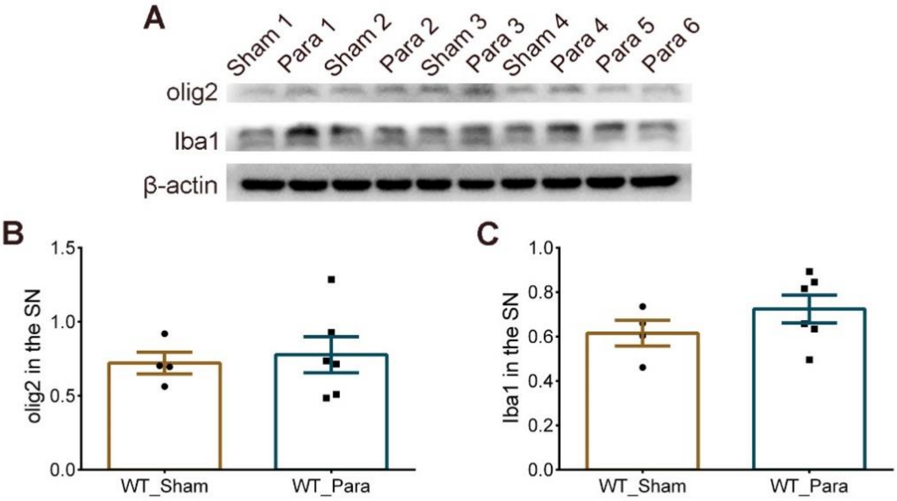


**Figure S1** The levels of Iba1 and olig2 in the SN of WT mice after parabiosis.

**(A,B)** No change was found in the levels of Iba1 and olig2 in the SN of WT mice after 4-month parabiosis, compared with WT-WT sham-operated group. (n=4, 6, the specific levels of each sample in different groups were shown as "•" and "▪").
